
JOURNAL INFORMATION
==============================
NLM Title Abbreviation: Hist Cienc Saude Manguinhos
Journal ID: Hist Cienc Saude Manguinhos
NLM Journal ID: 9513999
Journal ID: hcsm

História, Ciências, Saúde - Manguinhos

ISSN: 0104-5970
EISSN: 1678-4758
Publisher: Casa de Oswaldo Cruz - Fiocruz

ARTICLE INFORMATION
==============================
PMCID: PMC10425150
DOI: 10.1590/S0104-59702023000100037
Article ID: 01508
Article version: 1
Subjects: Resenhas

Devouring hope: global history connected

Devorando esperanças: a história global conectada

http://orcid.org/0000-0002-4582-4586 Silva Claiton i
i Universidade Federal da Fronteira Sul.Chapecó – SC – Brasil claiton@uffs.edu.br
Electronic publication date: 2023 Aug 11
Publication date: 2023
Volume: 30
Electronic Location ID: e2023037
Product: MCCOOK Stuart  
Coffee is not forever: a global history of the coffee leaf rust.
Athens: Ohio University Press. 2019. 281.
License: This is an Open Access article distributed under the terms of the Creative Commons Attribution License, which permits unrestricted use, distribution, and reproduction in any medium, provided the original work is properly cited.
License URL: https://creativecommons.org/licenses/by/4.0/

Figure count: 0
Table count: 0
Equation count: 0
Reference count: 3

==============================

O mais recente surto da chamada ferrugem do cafeeiro (*Hemileia Vastatrix*) se expandiu sobre fazendas, cobrindo territórios do Peru ao México durante a década de 2010, deixando árvores totalmente desfolhadas, embora vivas. A aparência de uma planta-esqueleto, sem folhas e, portanto, improdutivas comercialmente, espalhou-se tal qual a praga da ferrugem, gerando uma onda de prejuízos a pequenos e médios agricultores. Tendo essa pequena crônica-drama como ferramenta introdutória, o livro *Coffee is not forever: a global history of the coffee leaf rust* (McCook, 2019), do historiador canadense Stuart McCook, introduz e discute ao longo de dez capítulos como, desde seu primeiro registro, em 1869, no Ceilão (agora Sri Lanka), a *Hemileia Vastatrix* alcançou praticamente todos os cafezais do planeta, exceto os do Havaí, provocando múltiplos cenários (poucos deles animadores) para cientistas, produtores e governos. De certa forma, uma prévia dessa excelente narrativa já era conhecida por historiadores latino-americanos desde 2008, quando o autor publicou em português o artigo "Crônica de uma praga anunciada" (McCook, 2008). Somando-se aos artigos posteriores, McCook desenvolveu de forma bastante objetiva uma metodologia capaz de lidar com problemas da historiografia sobre epidemias agrícolas e as respostas da ciência sobre o assunto. Desde então, criou-se uma expectativa sobre como exatamente viria a ser o trabalho substituto do (já clássico) *States of nature* (McCook, 2002).

Para isso, o autor demonstra na primeira parte do livro como as epidemias agrícolas são possíveis apenas em condições que reúnam três elementos: um patógeno, um hospedeiro suscetível e, finalmente, condições ambientais favoráveis para a dispersão desse organismo. Historicamente, a emergência de *plantations* que se caracterizam por um alto número de indivíduos-plantas com baixa variação genética favoreceu surtos de epidemias como a ferrugem nos cafezais. De forma não intencional, esses esporos do patógeno foram carregados cada vez mais longe e mais rapidamente para zonas cafeeiras que antes nunca tiveram contato com o fungo. Portanto, se a intenção de governos, cientistas e produtores de café se orientava pela maximização da produtividade das emergentes *plantations*, a eclosão de epidemias como a ferrugem do cafeeiro criou novas orientações para a manipulação dos já mencionados três elementos causadores do surto no sentido de limitar sua expansão – utilizando instrumentos como quarentenas, produtos químicos ou criação de novas variedades de grãos mais resistentes. Um dos principais argumentos de McCook, nesse sentido, é indicar como *commodities* e suas doenças mudam dinâmicas da produção de *commodity* – por exemplo, consolidando o Brasil como um dos maiores produtores – e influenciando o surgimento de instituições bilaterais ou multilaterais voltadas para troca de saberes, tecnologias e germoplasmas. Em tempos de crise epidêmica, portanto, a imagem da Rainha Vermelha de Lewis Carroll serve para questionar certa ideia de progresso persistente na historiografia das ciências: "Produtores de café deviam correr mais rápido para permanecer no mesmo lugar" (McCook, 2019, p.8), em tradução livre, minimizando os danos.

Em geral, o livro se divide em três partes: a primeira demonstra como a onda inicial de ferrugem do cafeeiro durou quase um século, decisivamente moldado pelo colonialismo Europeu. O surto inicial de 1869 espalhou-se por meio das colônias tropicais europeias na Ásia, no Pacífico e na África. A conexão entre esses distantes cafezais foi preenchida pelos desígnios imperiais, enquanto a segunda fase (de metade da década de 1950 até meados da década de 1980) foi influenciada diretamente pela Guerra Fria. Finalmente, a fase neoliberal iniciou em 2008, com a *Big Rust*, na Colômbia e se espalhou pela América Central, em 2012, atingindo Porto Rico, México e Peru. A diferença dessa (até agora) última onda é que a *Big Rust* não foi impulsionada pela migração do patógeno para uma área previamente livre da doença; em parte, um complexo arranjo de mudança das condições meteorológicas favoreceu o fungo. As mudanças econômicas e institucionais no contexto da produção global do café também aumentaram a vulnerabilidade das *plantations* aos surtos epidêmicos.

Em termos historiográficos, argumenta McCook, enquanto um clássico relato das epidemias inicia com uma história dramática do primeiro surto e do caos subsequente, o autor defende que é preciso modificar o foco para plantas e ecossistemas. As epidemias, portanto, são produtos fundamentalmente históricos. Com isso, o historiador demonstra como a botânica e a ecologia do café – a partir do mais conhecido, o *Coffea arabica* – viajaram pelo mundo islâmico, onde sua base genética, portanto, provém de uma área livre de ferrugem. De uma planta cultivada por pequenos produtores do Ceilão, alcançou pequenos e médios produtores, até seu *boom* desde o século XIX.

O livro mais recente de McCook, obviamente, valeu a espera. Isso porque é um obra que traz muitas respostas aos historiadores da ciência, da tecnologia e do meio ambiente, especialmente sobre como lidar com contextos globais. Ou seja, se os chamados ambientes naturais não possuem fronteiras políticas, está mais do que na hora de os historiadores ambientais latino-americanos abraçarem metodologias, arquivos e questões de pesquisa que os levem a novos desafios. Deixando de lado o difusionismo e as ambivalências entre "centro" e "periferia" no que se refere à produção científica, *Coffee is not forever* demonstra como os saberes produzidos em cooperação transnacional criam “centros” móveis no Sul Global, caracterizados por uma fundamental troca de conhecimentos entre os atores, em resposta aos desafios impostos pelo patógeno e pelas mudanças ambientais.

A contribuição fundamental do livro de McCook é que, para além da intenção do autor, sua discussão abre uma possibilidade de pensar uma área disciplinar: de histórias conectadas entre o Sul Global. Explico: embora McCook tenha intencionado demonstrar a importância das trocas globais entre instituições científicas *et al.*, o livro descortina uma série de conexões não mediadas pelo Norte Global na transformação do ambiente e na tomada de decisões sobre as quais indústrias de *commodities* se fundamentam.


REFERÊNCIAS

MCCOOK Stuart Coffee is not forever: a global history of the coffee leaf rust Athens Ohio University Press 2019
MCCOOK Stuart Crônica de uma praga anunciada: epidemias agrícolas e história ambiental do café nas Américas Varia Historia 24 39 87 111 2008
MCCOOK Stuart States of nature: science, agriculture, and environment in the Spanish Caribbean, 1760-1940 Austin University of Texas Press 2002
